# spmodel: Spatial statistical modeling and prediction in R

**DOI:** 10.1371/journal.pone.0282524

**Published:** 2023-03-09

**Authors:** Michael Dumelle, Matt Higham, Jay M. Ver Hoef

**Affiliations:** 1 United States Environmental Protection Agency, Corvallis, Oregon, United States of America; 2 Department of Math, Computer Science, and Statistics, St. Lawrence University, Canton, New York, United States of America; 3 Marine Mammal Laboratory, National Oceanic and Atmospheric Administration Alaska Fisheries Science Center, Seattle, Washington, United States of America; Bangladesh Agricultural University, BANGLADESH

## Abstract

spmodel is an R package used to fit, summarize, and predict for a variety spatial statistical models applied to point-referenced or areal (lattice) data. Parameters are estimated using various methods, including likelihood-based optimization and weighted least squares based on variograms. Additional modeling features include anisotropy, non-spatial random effects, partition factors, big data approaches, and more. Model-fit statistics are used to summarize, visualize, and compare models. Predictions at unobserved locations are readily obtainable.

## Introduction

Spatial data are ubiquitous in everyday life and the scientific literature. As such, it is becoming increasingly important to properly analyze spatial data. Spatial data can be analyzed using a statistical model that explicitly incorporates the spatial dependence among nearby observations. Incorporating this spatial dependence can be challenging, but ignoring it often yields poor statistical models that incorrectly quantify uncertainty, impacting the validity of hypothesis tests, confidence intervals, and predictions intervals. spmodel provides tools to easily incorporate spatial dependence into statistical models, building upon commonly used R functions like lm().

spmodel implements model-based inference, which relies on fitting a statistical model. Model-based inference is different than design-based inference, which relies on random sampling and estimators that incorporate the properties of the random sample [1]. [2] defines two types of spatial data that can be analyzed using model-based inference: point-referenced data and areal data (areal data are sometimes called lattice data). Spatial data are point-referenced when they are observed at point-locations indexed by x-coordinates and y-coordinates on a spatially continuous surface with an infinite number of locations. Spatial models for point-referenced data are sometimes called geostatistical models. Spatial data are areal when they are observed as part of a finite network of polygons whose connections are indexed by a neighborhood structure. For example, the polygons may represent counties in a state who are neighbors if they share at least one boundary. Spatial models for areal data are sometimes called spatial autoregressive models. For thorough overviews of model-based inference in a spatial context, see [2–4].

Several R packages exist on CRAN that analyze either point-referenced or areal spatial data. For point-referenced data, they include fields [5], FRK [6], geoR [7], GpGp [8], gstat [9], LatticeKrig [10], R-INLA [11], rstan [12], spatial [13], spBayes [14], and spNNGP [15]. For areal data, they include brms [16], CARBayes [17], bigDM [18], and hglm [19]. Unlike these aforementioned packages, spmodel is designed to analyze both point-referenced and areal data using a common framework and syntax structure. spmodel also offers many features missing from the aforementioned R packages—together in one R package, spmodel offers detailed model summaries, extensive model diagnostics, non-spatial random effects, anisotropy, big data methods, prediction, the option to fix spatial covariance parameters at known values, and more.

The rest of this article is organized as follows. We first give a brief theoretical introduction to spatial linear models. We then outline the variety of methods used to estimate the parameters of spatial linear models. Next we explain how to obtain predictions at unobserved locations. Following that, we detail some advanced modeling features, including random effects, partition factors, anisotropy, and big data approaches. Finally we end with a short discussion.

Before proceeding, we install spmodel from CRAN and load it by running


R> install.packages(“spmodel”)
R> library(spmodel)


We create visualizations using ggplot2 [20], which we install from CRAN and load by running


R> install.packages(“ggplot2”)
R> library(ggplot2)


We also show code that can be used to create interactive visualizations of spatial data with mapview [21]. mapview has many backgrounds available that contextualize spatial data with topographical information. Before running the mapview code provided interactively, make sure that mapview is installed and loaded.

spmodel contains various methods for generic functions defined outside of spmodel. To find relevant documentation for these methods, run help(“generic.spmodel”, “spmodel”) (e.g., help(“fitted.spmodel”, “spmodel”), help(“summary.spmodel”, “spmodel”), help(“plot.spmodel”, “spmodel”), help(“predict.spmodel”, “spmodel”), help(“tidy.spmodel”, “spmodel”), etc.). We provide more details and examples regarding these methods and generics throughout this vignette. For a full list of spmodel functions available, see spmodel’s documentation manual.

## The spatial linear model

Statistical linear models are often parameterized as

$$\begin{array}{c} y=X\beta +\epsilon , \end{array}$$ (1)

where for a sample size *n*, **y** is an *n* × 1 column vector of response variables, **X** is an *n* × *p* design (model) matrix of explanatory variables, ***β*** is an *p* × 1 column vector of fixed effects controlling the impact of **X** on **y**, and ***ϵ*** is an *n* × 1 column vector of random errors. We typically assume that E(***ϵ***) = **0** and $\text{Cov}\left(\epsilon \right)=\sigma_{\epsilon }^{2}I$, where E(⋅) denotes expectation, Cov(⋅) denotes covariance, $\sigma_{\epsilon }^{2}$ denotes a variance parameter, and **I** denotes the identity matrix.

The model in Eq 1 assumes the elements of **y** are uncorrelated. Typically for spatial data, elements of **y** are correlated, as observations close together in space tend to be more similar than observations far apart [22]. Failing to properly accommodate the spatial dependence in **y** can lead researchers to incorrect conclusions about their data. To accommodate spatial dependence in **y**, an *n* × 1 spatial random effect, ***τ***, is added to Eq 1, yielding the model

$$\begin{array}{c} y=X\beta +\tau +\epsilon , \end{array}$$ (2)

where ***τ*** is independent of ***ϵ***, E(***τ***) = **0**, $\text{Cov}\left(\tau \right)=\sigma_{\tau }^{2}R$, and **R** is a matrix that determines the spatial dependence structure in **y** and depends on a range parameter, *φ*. We discuss **R** in more detail shortly. The parameter $\sigma_{\tau }^{2}$ is called the spatially dependent random error variance or partial sill. The parameter $\sigma_{\epsilon }^{2}$ is called the spatially independent random error variance or nugget. These two variance parameters are henceforth more intuitively written as $\sigma_{de}^{2}$ and $\sigma_{ie}^{2}$, respectively. The covariance of **y** is denoted **Σ** and given by $\sigma_{de}^{2}R+\sigma_{ie}^{2}I$. The parameters that compose this covariance are contained in the vector ***θ***, which is called the covariance parameter vector.

Eq 2 is called the spatial linear model. The spatial linear model applies to both point-referenced and areal data. The splm() function is used to fit spatial linear models for point-referenced data (i.e., geostatistical models). One spatial covariance function available in splm() is the exponential spatial covariance function, which has an **R** matrix given by

$$\begin{array}{c} R=\text{exp}(-M/\phi ), \end{array}$$ (3)

where **M** is a matrix of Euclidean distances among observations. Recall that *φ* is the range parameter, and it controls the behavior of **R** as a function of distance. In Eq 3, as the distance between two observations increases, the correlation between them decreases. Parameterizations for other splm() spatial covariance types and their **R** matrices can be viewed by running help(“splm”, “spmodel”) or vignette(“technical”, “spmodel”). Some of these spatial covariance types (e.g., Matérn) depend on an extra parameter beyond $\sigma_{de}^{2}$, $\sigma_{ie}^{2}$, and *ϕ*.

The spautor() function is used to fit spatial linear models for areal data (i.e., spatial autoregressive models). One spatial autoregressive covariance function available in spautor() is the simultaneous autoregressive spatial covariance function, which has an **R** matrix given by

$$\begin{array}{c} R={\left[\left(I-\phi W\right){\left(I-\phi W\right)}^{⊤}\right]}^{-1}, \end{array}$$

where **W** is a weight matrix describing the neighborhood structure in **y**. Parameterizations for spautor() spatial covariance types and their **R** matrices can be seen by running help(“spautor”, “spmodel”) or vignette(“technical”, “spmodel”).

One way to define **W** is through queen contiguity [23]. Two observations are queen contiguous if they share a boundary. The *ij*th element of **W** is then one if observation *i* and observation *j* are queen contiguous and zero otherwise. Observations are not considered neighbors with themselves, so each diagonal element of **W** is zero.

Sometimes each element in the weight matrix **W** is divided by its respective row sum. This is called row-standardization. Row-standardizing **W** has several benefits, which are discussed in detail by [24].

## Model fitting

In this section, we show how to use the splm() and spautor() functions to estimate parameters of the spatial linear model. We also explore diagnostic tools in spmodel that evaluate model fit. The splm() and spautor() functions share similar syntactic structure with the lm() function used to fit non-spatial linear models from Eq 1. splm() and spautor() generally require at least three arguments:

- formula: a formula that describes the relationship between the response variable (**y**) and explanatory variables (**X**)
  – formula in splm() is the same as formula in lm()
- data: a data.frame or sf object that contains the response variable, explanatory variables, and spatial information
- spcov_type: the spatial covariance type (“exponential”, “matern”, “car”, etc)

If data is an sf [25] object, spatial information is stored in the object’s geometry. If data is a data.frame, then the x-coordinates and y-coordinates must be provided via the xcoord and ycoord arguments (for point-referenced data) or the weight matrix must be provided via the W argument (for areal data).

In the following subsections, we use the point-referenced moss data, an sf object that contains data on heavy metals in mosses near a mining road in Alaska. We view the first few rows of moss by running


R> moss

Simple feature collection with 365 features and 7 fields
Geometry type: POINT
Dimension:     XY
Bounding box:  xmin: −445884.1 ymin: 1929616 xmax: −383656.8 ymax: 2061414
Projected CRS: NAD83 / Alaska Albers
# A tibble: 365 x 8
   sample field_dup lab_rep year  sideroad log_dist2road log_Zn
   <fct>  <fct>     <fct>   <fct> <fct>            <dbl>  <dbl>
 1 001PR  1         1       2001  N                 2.68   7.33
 2 001PR  1         2       2001  N                 2.68   7.38
 3 002PR  1         1       2001  N                 2.54   7.58
 4 003PR  1         1       2001  N                 2.97   7.63
 5 004PR  1         1       2001  N                 2.72   7.26
 6 005PR  1         1       2001  N                 2.76   7.65
 7 006PR  1         1       2001  S                 2.30   7.59
 8 007PR  1         1       2001  N                 2.78   7.16
 9 008PR  1         1       2001  N                 2.93   7.19
10 009PR  1         1       2001  N                 2.79   8.07
# … with 355 more rows, and 1 more variable:
#   geometry <POINT [m]>


**Fig 1:**
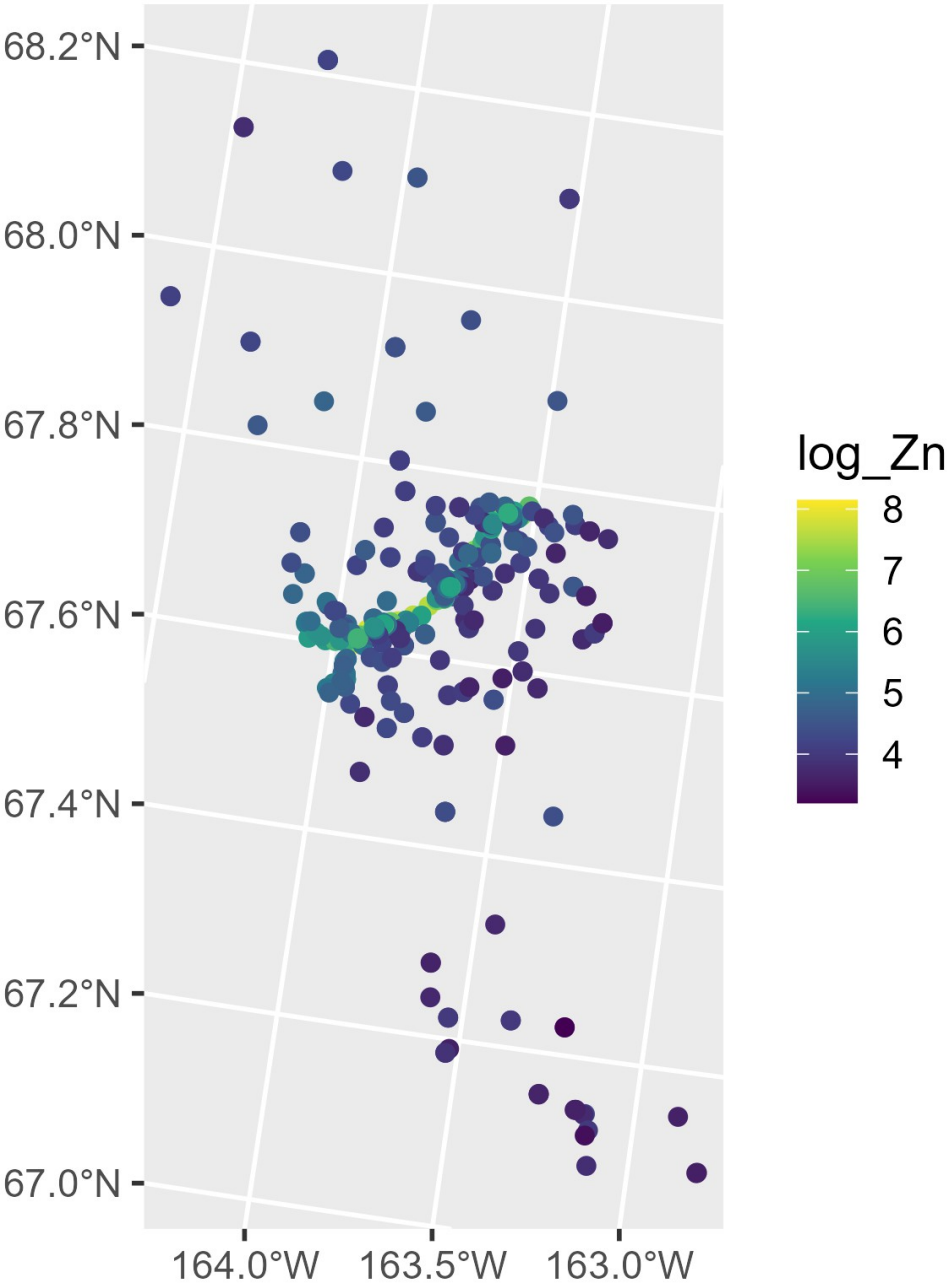
Distribution of log zinc concentration in the moss data.

We can learn more about moss by running help(“moss”, “spmodel”), and we can visualize the distribution of log zinc concentration in moss (Fig 1) by running


R> ggplot(moss, aes(color = log_Zn)) +
+   geom_sf(size = 2) +
+   scale_color_viridis_c() +
+   theme_gray(base_size = 14)


Log zinc concentration can be viewed interactively in mapview by running


R> mapview(moss, zcol = “log_Zn”)


### Estimation

Generally the covariance parameters (***θ***) and fixed effects (***β***) of the spatial linear model require estimation. The default estimation method in spmodel is restricted maximum likelihood [26–28]. Maximum likelihood estimation is also available. For point-referenced data, semivariogram weighted least squares [29] and semivariogram composite likelihood [30] are additional estimation methods. The estimation method is chosen using the estmethod argument.

We estimate parameters of a spatial linear model regressing log zinc concentration (log_Zn) on log distance to a haul road (log_dist2road) using an exponential spatial covariance function by running


R> spmod <- splm(log_Zn ^~^ log_dist2road, moss, spcov_type = “exponential”)


We summarize the model fit by running


R> summary(spmod)

Call:
splm(formula = log_Zn ^~^ log_dist2road, data = moss, spcov_type = “exponential”)

Residuals:
    Min      1Q  Median      3Q     Max 
−2.6801 −1.3606 −0.8103 −0.2485  1.1298 

Coefficients (fixed):
              Estimate Std. Error z value Pr(>|z|)    
(Intercept)    9.76825    0.25216   38.74   <2e–16 ***
log_dist2road −0.56287    0.02013  −27.96   <2e–16 ***
---
Signif. codes:  0 ‘***’ 0.001 ‘**’ 0.01 ‘*’ 0.05 ‘.’ 0.1 ‘ ’ 1

Pseudo R-squared: 0.683

Coefficients (exponential spatial covariance):
       de        ie     range 
3.595e-01 7.897e-02 8.237e+03 


The fixed effects coefficient table contains estimates, standard errors, z-statistics, and asymptotic p-values for each fixed effect. From this table, we notice there is evidence that mean log zinc concentration significantly decreases with distance from the haul road (p-value < 2e-16). We see the fixed effect estimates by running


R> coef(spmod)

  (Intercept) log_dist2road 
    9.7682525    −0.5628713 


The model summary also contains the exponential spatial covariance parameter estimates, which we can view by running


R> coef(spmod, type = “spcov”)
          de           ie        range       rotate        scale 
3.595316e-01 7.896824e-02 8.236712e+03 0.000000e+00 1.000000e+00 
attr(,“class”)
[1] “exponential”


**Fig 2:**
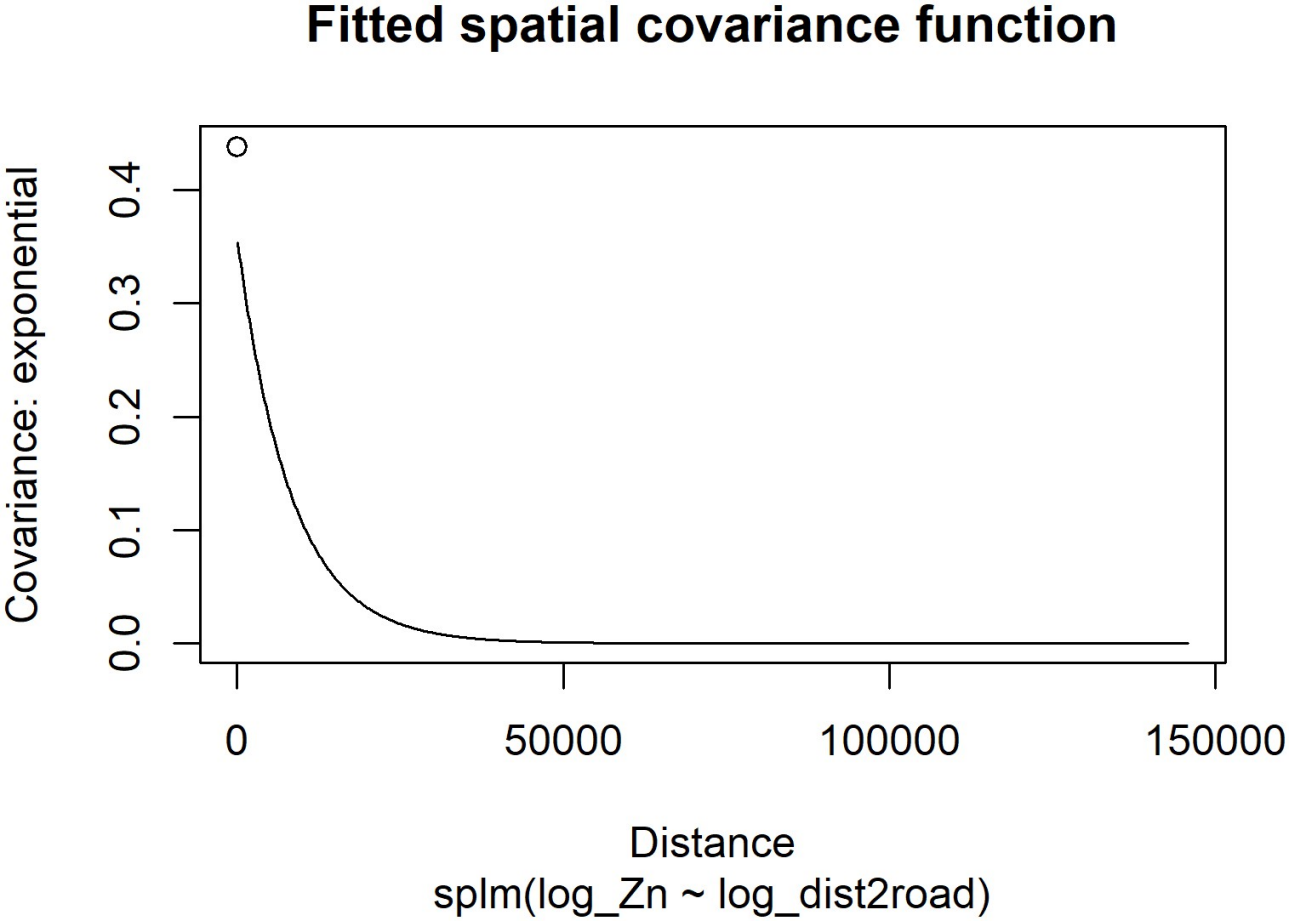
Empirical spatial covariance of the fitted model. The open circle at a distance of zero represents the $\sigma_{de}^{2}+\sigma_{ie}^{2}$. The solid line at positive distances represents $\sigma_{de}^{2}R$ at a particular distance.

The dependent random error variance ($\sigma_{de}^{2}$) is estimated to be approximately 0.36 and the independent random error variance ($\sigma_{ie}^{2}$) is estimated to be approximately 0.079. The range (*ϕ*) is estimated to be approximately 8,237. The effective range is the distance at which the spatial covariance is approximately zero. For the exponential covariance, the effective range is 3*ϕ*. This means that observations whose distance is greater than 24,711 meters are approximately uncorrelated. The rotate and scale parameters affect the modeling of anisotropy, which we discuss later. By default, rotate and scale are assumed to be zero and one, respectively, which means that anisotropy is not modeled (i.e., the spatial covariance is assumed isotropic, or independent of direction). We visualize the fitted spatial covariance function (Fig 2) by running


R> plot(spmod, which = 7)


### Model-fit statistics

The quality of model fit can be assessed using a variety of statistics readily available in spmodel. The first model-fit statistic we consider is the pseudo R-squared. The pseudo R-squared is a generalization of the classical R-squared from non-spatial linear models that quantifies the proportion of variability in the data explained by the fixed effects. The pseudo R-squared is defined as

$$\begin{array}{c} PR2=1-\frac{D_{\hat{\beta }}}{D_{\hat{\mu }}}, \end{array}$$

where $D_{\hat{\beta }}$ is the deviance of the fitted model with all explanatory variables and $D_{\hat{\beta }}$ is the deviance of the fitted model with only an intercept. We compute the pseudo R-squared by running


R> pseudoR2(spmod)
[1] 0.6829687


Roughly 68% of the variability in log zinc is explained by log distance from the road. The pseudo R-squared can be adjusted to account for the number of explanatory variables using the adjust argument. Pseudo R-squared (and the adjusted version) is most helpful for comparing models that have the same covariance structure.

The next two model-fit statistics we consider are the AIC and AICc that [31] derive for spatial data. The AIC and AICc evaluate the fit of a model with a penalty for the number of parameters estimated. This penalty balances model fit and model parsimony. Lower AIC and AICc indicate a better balance of model fit and parsimony. The AICc is a correction to AIC that is better suited for small sample sizes. As the sample size increases, AIC and AICc converge.

The AIC and AICc are given by

$$\begin{array}{c} \begin{array}{cc} \text{AIC} & =-2\ell \left(\hat{\Theta }\right)+2(|\hat{\Theta }\left|\right)\\ \text{AICc} & =-2\ell \left(\hat{\Theta }\right)+2n(|\hat{\Theta }\left|)/(n-\right|\hat{\Theta }\left|-1\right), \end{array} \end{array}$$

where $\ell (\hat{\Theta })$ is the log-likelihood of the data evaluated at the estimated parameter vector $\hat{\Theta }$ that maximized *ℓ*(**Θ**), $|\hat{\Theta }|$ is the cardinality of $\hat{\Theta }$, and *n* is the sample size. For maximum likelihood, $\hat{\Theta }=\left\{\hat{\Theta },\hat{\beta }\right\}$, and for restricted maximum likelihood, $\hat{\Theta }=\left\{\hat{\Theta }\right\}$. There are some nuances to consider when comparing AIC across models: AIC comparisons between a model fit using restricted maximum likelihood and a model fit using maximum likelihood are meaningless, as the models are fit with different likelihoods; and AIC comparisons between models fit using restricted maximum likelihood are only valid when the models have the same fixed effect structure; AIC comparisons between models fit using maximum likelihood are valid even when the models have different fixed effect structures [32].

Suppose we want to quantify the difference in model quality between the spatial model and a non-spatial model using the AIC and AICc criteria. We fit a non-spatial model (Eq 1) in spmodel by running


R> lmod <- splm(log_Zn ^~^ log_dist2road, moss, spcov_type = “none”)


This model is equivalent to one fit using lm(). We compute the spatial AIC and AICc of the spatial model and non-spatial model by running


R> AIC(spmod, lmod)

      df      AIC
spmod  3 373.2089
lmod   1 636.0635

R> AICc(spmod, lmod)

      df     AICc
spmod  3 373.2754
lmod   1 636.0745


The noticeably lower AIC and AICc of of the spatial model indicate that it is a better fit to the data than the non-spatial model. Recall that these AIC and AICc comparisons are valid because both models are fit using restricted maximum likelihood (the default).

Another approach to comparing the fitted models is to perform leave-one-out cross validation [33]. In leave-one-out cross validation, a single observation is removed from the data, the model is re-fit, and a prediction is made for the held-out observation. Then, a loss metric like mean-squared-prediction error is computed and used to evaluate model fit. The lower the mean-squared-prediction error, the better the model fit. For computational efficiency, leave-one-out cross validation in spmodel is performed by first estimating ***θ*** using all the data and then re-estimating ***β*** for each observation. We perform leave-one-out cross validation for the spatial and non-spatial model by running


R> loocv(spmod)

[1] 0.1110895

R> loocv(lmod)

[1] 0.3237897


The noticeably lower mean-squared-prediction error of the spatial model indicates that it is a better fit to the data than the non-spatial model.

### Diagnostics

In addition to model fit metrics, spmodel provides functions to compute diagnostic metrics that help assess model assumptions and identify unusual observations.

An observation is said to have high leverage if its combination of explanatory variable values is far from the mean vector of the explanatory variables. For a non-spatial model, the leverage of the *i*th observation is the *i*th diagonal element of the hat matrix given by

$$\begin{array}{c} H=X{\left(X^{⊤}X\right)}^{-1}X^{⊤}. \end{array}$$

For a spatial model, the leverage of the *i*th observation is the *i*th diagonal element of the spatial hat matrix given by

$$\begin{array}{c} H^{*}=\left(X^{*}{\left(X^{*⊤}X\right)}^{-1}X^{*⊤}\right), \end{array}$$

where X* = **Σ**^−1/2^**X** and **Σ**^−1/2^ is the inverse square root of the covariance matrix, **Σ** [34]. The spatial hat matrix can be viewed as the non-spatial hat matrix applied to X* instead of **X**. We compute the hat values (leverage) by running


R> hatvalues(spmod)


Larger hat values indicate more leverage, and observations with large hat values may be unusual and warrant further investigation.

The fitted value of an observation is the estimated mean response given the observation’s explanatory variable values and the model fit:

$$\begin{array}{c} \hat{y}=X\hat{\beta }. \end{array}$$

We compute the fitted values by running


R> fitted(spmod)


Fitted values for the spatially dependent random errors (***τ***), spatially independent random errors (***ϵ***), and random effects can also be obtained via fitted() by changing the type argument.

The residuals measure each response’s deviation from its fitted value. The response residuals are given by

$$\begin{array}{c} e_{r}=y-\hat{y}. \end{array}$$

We compute the response residuals of the spatial model by running


R> residuals(spmod)


The response residuals are typically not directly checked for linear model assumptions, as they have covariance closely resembling the covariance of **y**. Pre-multiplying the residuals by **Σ**^−1/2^ yields the Pearson residuals [35]:

$$\begin{array}{c} e_{p}=\Sigma^{-1/2}e_{r}. \end{array}$$

When the model is correct, the Pearson residuals have mean zero, variance approximately one, and are uncorrelated. We compute the Pearson residuals of the spatial model by running


R> residuals(spmod, type = “pearson”)


The covariance of **e**_*p*_ is (**I** − H*), which is approximately **I** for large sample sizes. Explicitly dividing **e**_*p*_ by the respective diagonal element of (**I** − H*) yields the standardized residuals [35]:

$$\begin{array}{c} e_{s}=\frac{e_{p}}{\sqrt{\left(1-\text{diag}\left(H^{*}\right)\right)}}, \end{array}$$

where diag(H*) denotes the diagonal of H*. We compute the standardized residuals of the spatial model by running


R> residuals(spmod, type = “standardized”)


or


R> rstandard(spmod)


When the model is correct, the standardized residuals have mean zero, variance one, and are uncorrelated.

It is common to check linear model assumptions through visualizations. We can visualize the standardized residuals vs fitted values by running


R> plot(spmod, which = 1) # figure omitted


When the model is correct, the standardized residuals should be evenly spread around zero with no discernible pattern. We can visualize a normal QQ-plot of the standardized residuals by running


R> plot(spmod, which = 2) # figure omitted


When the standardized residuals are normally distributed, they should closely follow the normal QQ-line.

An observation is said to be influential if its omission has a large impact on model fit. Typically, this is measured using Cook’s distance [36]. For the non-spatial model, the Cook’s distance of the *i*th observation is denoted **D** and given by

$$\begin{array}{c} D=e_{s}^{2}\frac{\text{diag}(H)}{p(1-\text{diag}(H))}, \end{array}$$

where *p* is the dimension of ***β*** (the number of fixed effects).

For a spatial model, the Cook’s distance of the *i*th observation is denoted D* and given by

$$\begin{array}{c} D^{*}=e_{s}^{2}\frac{\text{diag}(H^{*})}{p(1-\text{diag}\left(H^{*}\right))}. \end{array}$$

A larger Cook’s distance indicates more influence, and observations with large Cook’s distance values may be unusual and warrant further investigation. We compute Cook’s distance by running


R> cooks.distance(spmod)


The Cook’s distance versus leverage (hat values) can be visualized by running


R> plot(spmod, which = 6) # figure omitted


Though we described the model diagnostics in this subsection using **Σ**, generally the covariance parameters are estimated and **Σ** is replaced with $\hat{\Sigma }$.

### The broom functions: tidy(), glance(), and augment()

The tidy(), glance(), and augment() functions from the broom R package [37] provide convenient output for many of the model fit and diagnostic metrics discussed in the previous two sections. The tidy() function returns a tidy tibble of the coefficient table from summary():


R> tidy(spmod)

# A tibble: 2 x 5
  term          estimate std.error statistic p.value
  <chr>            <dbl>     <dbl>     <dbl>   <dbl>
1 (Intercept)      9.77     0.252       38.7       0
2 log_dist2road   −0.563    0.0201     −28.0       0


This tibble format makes it easy to pull out the coefficient names, estimates, standard errors, z-statistics, and p-values from the summary() output.

The glance() function returns a tidy tibble of model-fit statistics:


R> glance(spmod)

# A tibble: 1 x 9
      n     p  npar value   AIC  AICc logLik deviance pseudo.r.squared
  <int> <dbl> <int> <dbl> <dbl> <dbl>  <dbl>    <dbl>            <dbl>
1   365     2     3  367.  373.  373.  −184.      363            0.683


The glances() function is an extension of glance() that can be used to look at many models simultaneously:


R> glances(spmod, lmod)

# A tibble: 2 x 10
  model     n     p  npar value   AIC  AICc logLik deviance
  <chr> <int> <dbl> <int> <dbl> <dbl> <dbl>  <dbl>    <dbl>
1 spmod   365     2     3  367.  373.  373.  −184.     363 
2 lmod    365     2     1  634.  636.  636.  −317.     363.
# … with 1 more variable: pseudo.r.squared <dbl>


Finally, the augment() function augments the original data with model diagnostics:


R> augment(spmod)

Simple feature collection with 365 features and 7 fields
Geometry type: POINT
Dimension:     XY
Bounding box:  xmin: −445884.1 ymin: 1929616 xmax: −383656.8 ymax: 2061414
Projected CRS: NAD83 / Alaska Albers
# A tibble: 365 x 8
   log_Zn log_dist2road .fitted .resid    .hat  .cooksd .std.resid
 *  <dbl>         <dbl>   <dbl>  <dbl>   <dbl>    <dbl>      <dbl>
 1   7.33          2.68    8.26 −0.928 0.102   0.112        −1.48 
 2   7.38          2.68    8.26 −0.880 0.0101  0.000507     −0.316
 3   7.58          2.54    8.34 −0.755 0.0170  0.000475     −0.236
 4   7.63          2.97    8.09 −0.464 0.0137  0.000219      0.178
 5   7.26          2.72    8.24 −0.977 0.0177  0.00515      −0.762
 6   7.65          2.76    8.21 −0.568 0.0147  0.000929     −0.355
 7   7.59          2.30    8.47 −0.886 0.0170  0.00802      −0.971
 8   7.16          2.78    8.20 −1.05  0.0593  0.0492       −1.29 
 9   7.19          2.93    8.12 −0.926 0.00793 0.000451     −0.337
10   8.07          2.79    8.20 −0.123 0.0265  0.00396       0.547
# … with 355 more rows, and 1 more variable: geometry <POINT [m]>


By default, only the columns of data used to fit the model are returned alongside the diagnostics. All original columns of data are returned by setting drop to FALSE. augment() is especially powerful when the data are an sf object because model diagnostics can be easily visualized spatially. For example, we could subset the augmented object so that it only includes observations whose standardized residuals have absolute values greater than some cutoff and then map them.

### An areal data example

Next we use the seal data, an sf object that contains the log of the estimated harbor-seal trends from abundance data across polygons in Alaska, to provide an example of fitting a spatial linear model for areal data using spautor(). We view the first few rows of seal by running


R> seal

Simple feature collection with 62 features and 1 field
Geometry type: POLYGON
Dimension:     XY
Bounding box:  xmin: 913618.8 ymin: 1007542 xmax: 1116002 ymax: 1145054
Projected CRS: NAD83 / Alaska Albers
# A tibble: 62 x 2
   log_trend                    geometry
       <dbl>               <POLYGON [m]>
 1  NA       ((1035002 1054710, 1035002^~^
 2  −0.282   ((1037002 1039492, 1037006^~^
 3  −0.00121 ((1070158 1030216, 1070185^~^
 4   0.0354  ((1054906 1034826, 1054931^~^
 5  −0.0160  ((1025142 1056940, 1025184^~^
 6   0.0872  ((1026035 1044623, 1026037^~^
 7  −0.266   ((1100345 1060709, 1100287^~^
 8   0.0743  ((1030247 1029637, 1030248^~^
 9  NA       ((1043093 1020553, 1043097^~^
10  −0.00961 ((1116002 1024542, 1116002^~^
# … with 52 more rows


We can learn more about the data by running help(“seal”, “spmodel”).

**Fig 3:**
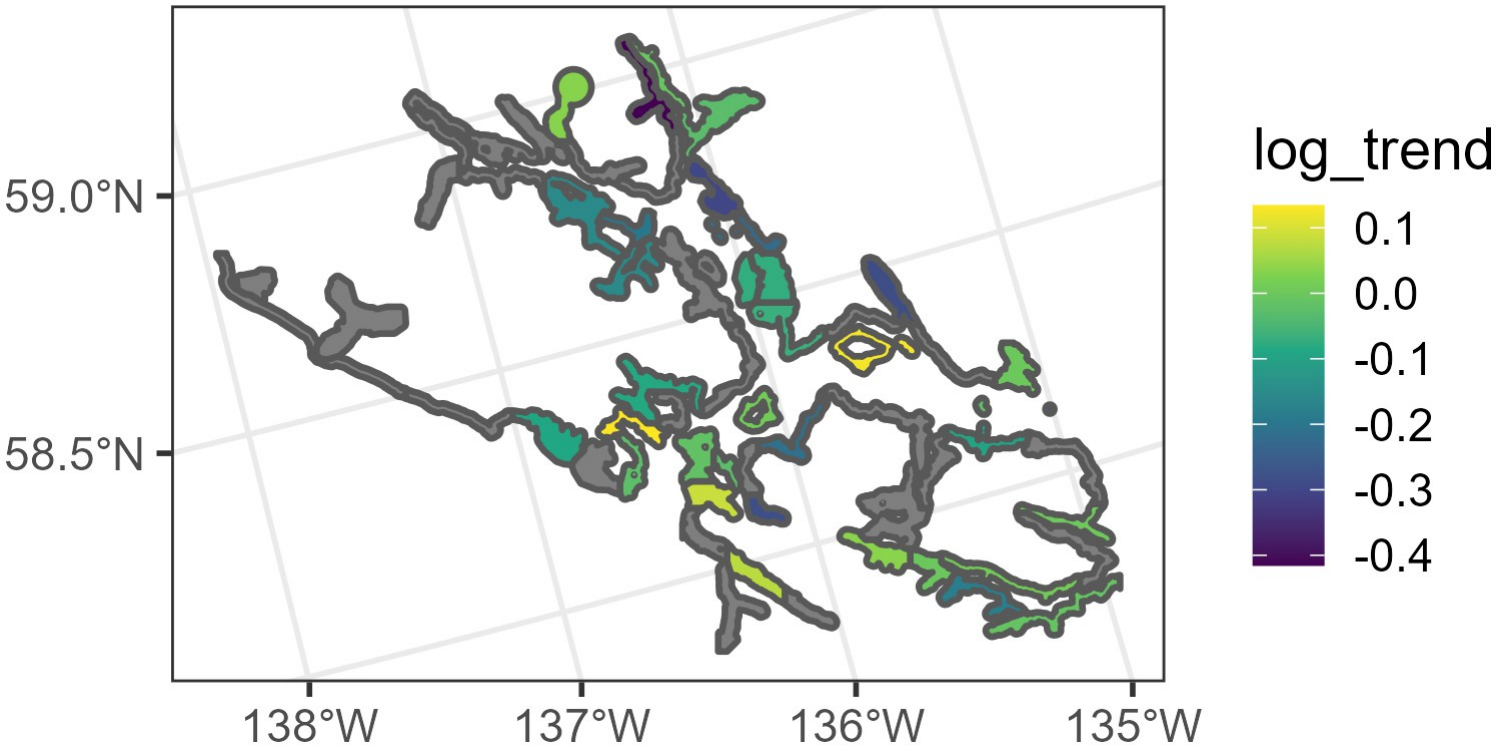
Distribution of log seal trends in the seal data. Polygons are gray if seal trends are missing.

We can visualize the distribution of log seal trends in the seal data (Fig 3) by running


R> ggplot(seal, aes(fill = log_trend)) +
+   geom_sf(size = 0.75) +
+   scale_fill_viridis_c() +
+   theme_bw(base_size = 14)


Log trends can be viewed interactively in mapview by running


R> mapview(seal, zcol = “log_trend”)


The gray polygons denote areas where the log trend is missing. These missing areas need to be kept in the data while fitting the model to preserve the overall neighborhood structure.

We estimate parameters of a spatial autoregressive model for log seal trends (log_trend) using an intercept-only model with a conditional autoregressive (CAR) spatial covariance by running


R> sealmod <- spautor(log_trend ^~^ 1, seal, spcov_type = “car”)


If a weight matrix is not provided to spautor(), it is calculated internally using queen contiguity. Recall that queen contiguity defines two observations as neighbors if they share at least one common boundary. If at least one observation has no neighbors, the extra parameter is estimated, which quantifies variability among observations without neighbors. By default, spautor() uses row standardization [24] and assumes an independent error variance (ie) of zero.

We summarize, tidy, glance at, and augment the fitted model by running


R> summary(sealmod)

Call:
spautor(formula = log_trend ^~^ 1, data = seal, spcov_type = “car”)

Residuals:
     Min       1Q   Median       3Q      Max 
−0.34443 −0.10405  0.04422  0.07349  0.20487 

Coefficients (fixed):
            Estimate Std. Error z value Pr(>|z|)   
(Intercept) −0.07102    0.02495  −2.846  0.00443 **
---
Signif. codes:  
0 ‘***’ 0.001 ‘**’ 0.01 ‘*’ 0.05 ‘.’ 0.1 ‘ ’ 1

Coefficients (car spatial covariance):
     de   range   extra 
0.03261 0.41439 0.02221 

R> tidy(sealmod)

# A tibble: 1 x 5
  term        estimate std.error statistic p.value
  <chr>          <dbl>     <dbl>     <dbl>   <dbl>
1 (Intercept)  −0.0710    0.0250     −2.85 0.00443

R> glance(sealmod)

# A tibble: 1 x 9
      n     p  npar value   AIC  AICc logLik deviance pseudo.r.squared
  <int> <dbl> <int> <dbl> <dbl> <dbl>  <dbl>    <dbl>            <dbl>
1    34     1     3 −36.9 −30.9 −30.1   18.4     32.9                0

R> augment(sealmod)

Simple feature collection with 34 features and 6 fields
Geometry type: POLYGON
Dimension:     XY
Bounding box:  xmin: 980001.5 ymin: 1010815 xmax: 1116002 ymax: 1145054
Projected CRS: NAD83 / Alaska Albers
# A tibble: 34 x 7
   log_trend .fitted  .resid   .hat .cooksd .std.resid
 *     <dbl>   <dbl>   <dbl>  <dbl>   <dbl>      <dbl>
 1  −0.282   −0.0710 −0.211  0.0179 0.0233      −1.14 
 2  −0.00121 −0.0710  0.0698 0.0699 0.0412       0.767
 3   0.0354  −0.0710  0.106  0.0218 0.0109       0.705
 4  −0.0160  −0.0710  0.0550 0.0343 0.00633      0.430
 5   0.0872  −0.0710  0.158  0.0229 0.0299       1.14 
 6  −0.266   −0.0710 −0.195  0.0280 0.0493      −1.33 
 7   0.0743  −0.0710  0.145  0.0480 0.0818       1.30 
 8  −0.00961 −0.0710  0.0614 0.0143 0.00123      0.293
 9  −0.182   −0.0710 −0.111  0.0131 0.0155      −1.09 
10   0.00351 −0.0710  0.0745 0.0340 0.0107       0.561
# … with 24 more rows, and 1 more variable:
#   geometry <POLYGON [m]>


Note that for spautor() models, the ie spatial covariance parameter is assumed zero by default (and omitted from the summary() output). This default behavior can be overridden by specifying ie in the spcov_initial argument to spautor(). Also note that the pseudo R-squared is zero because there are no explanatory variables in the model (i.e., it is an intercept-only model).

## Prediction

In this section, we show how to use predict() to perform spatial prediction (also called Kriging) in spmodel. We will fit a model using the point-referenced sulfate data, an sf object that contains sulfate measurements in the conterminous United States, and make predictions for each location in the point-referenced sulfate_preds data, an sf object that contains locations in the conterminous United States at which to predict sulfate.

**Fig 4:**
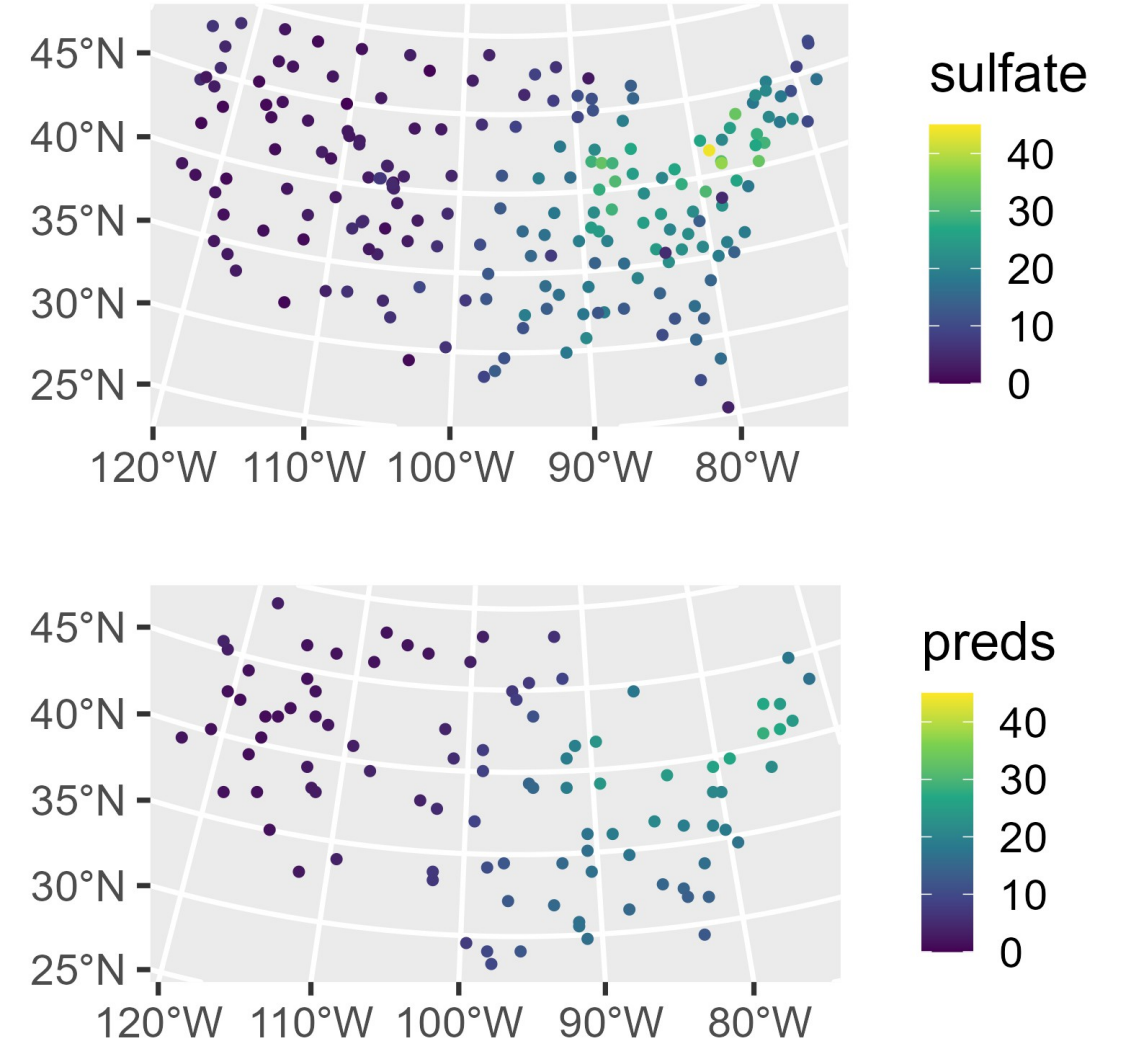
Distribution of observed sulfate and sulfate predictions in the conterminous United States. In A (top), observed sulfate is visualized. In B (bottom), sulfate predictions are visualized.

We first visualize the distribution of the sulfate data (Fig 4A) by running


R> ggplot(sulfate, aes(color = sulfate)) +
+   geom_sf(size = 2.5) +
+   scale_color_viridis_c(limits = c(0, 45)) +
+   theme_gray(base_size = 18)


We then fit a spatial linear model for sulfate using an intercept-only model with a spherical spatial covariance function by running


R> sulfmod <- splm(sulfate ^~^ 1, sulfate, spcov_type = “spherical”)


Then we obtain best linear unbiased predictions (Kriging predictions) using predict(). The newdata argument contains the locations at which to predict, and we store the predictions as a new variable in sulfate_preds called preds by running


R> sulfate_preds$preds <- predict(sulfmod, newdata = sulfate_preds)


We can visualize the model predictions (Fig 4B) by running


R> ggplot(sulfate_preds, aes(color = preds)) +
+   geom_sf(size = 2.5) +
+   scale_color_viridis_c(limits = c(0, 45)) +
+   theme_gray(base_size = 18)


It is important to properly specify the newdata object when running predict(). If explanatory variables were used to fit the model, the same explanatory variables must be included in newdata with the same names as they have in data. Additionally, if an explanatory variable is categorical or a factor, the values of this variable in newdata must also be values in data (e.g., if a categorical variable with values “A”, and “B” was used to fit the model, the corresponding variable in newdata cannot have a value “C”). If data is a data.frame, coordinates must be included in newdata with the same names as they have in data. If data is an sf object, coordinates must be included in newdata with the same geometry name as they have in data. When using projected coordinates, the projection for newdata should be the same as the projection for data.

Prediction standard errors are returned by setting the se.fit argument to TRUE:


R> predict(sulfmod, newdata = sulfate_preds, se.fit = TRUE)


The interval argument determines the type of interval returned. If interval is “none” (the default), no intervals are returned. If interval is “prediction”, then 100 * level% prediction intervals are returned (the default is 95% prediction intervals):


R> predict(sulfmod, newdata = sulfate_preds, interval = “prediction”)


If interval is “confidence”, the predictions are instead the estimated mean given each observation’s explanatory variable values (i.e., fitted values) and the corresponding 100 * level% confidence intervals are returned:


R> predict(sulfmod, newdata = sulfate_preds, interval = “confidence”)


The predict() output structure changes based on interval and se.fit. For more details, run help(“predict.spmodel”, “spmodel”).

Previously we used the augment() function to augment data with model diagnostics. We can also use augment() to augment newdata with predictions, standard errors, and intervals. We remove the model predictions from sulfate_preds before showing how augment() is used to obtain the same predictions by running


R> sulfate_preds$preds <- NULL


We then view the first few rows of sulfate_preds augmented with a 90% prediction interval by running


R> augment(
+   sulfmod,
+   newdata = sulfate_preds,
+   interval = “prediction”,
+   level = 0.90
+ )
Simple feature collection with 100 features and 3 fields
Geometry type: POINT
Dimension:     XY
Bounding box:  xmin: −2283774 ymin: 582930.5 xmax: 1985906 ymax: 3037173
Projected CRS: NAD83 / Conus Albers
# A tibble: 100 x 4
   .fitted .lower .upper            geometry
 *   <dbl>  <dbl>  <dbl>         <POINT [m]>
 1    1.40  −5.33   8.14  (−1771413 1752976)
 2   24.5   18.2   30.8    (1018112 1867127)
 3    8.99   2.36  15.6  (−291256.8 1553212)
 4   16.4    9.92  23.0    (1274293 1267835)
 5    4.91  −1.56  11.4  (−547437.6 1638825)
 6   26.7   20.4   33.0    (1445080 1981278)
 7    3.00  −3.65   9.66  (−1629090 3037173)
 8   14.3    7.97  20.6    (1302757 1039534)
 9    1.49  −5.08   8.06  (−1429838 2523494)
10   14.4    7.97  20.8    (1131970 1096609)
# … with 90 more rows


Here .fitted represents the predictions, .lower represents the lower bound of the 90% prediction intervals, and .upper represents the upper bound of the 90% prediction intervals.

An alternative (but equivalent) approach can be used for model fitting and prediction that circumvents the need to keep data and newdata as separate objects. Suppose that observations requiring prediction are stored in data as missing (NA) values. We can add a column of missing values to sulfate_preds and then bind it together with sulfate by running


R> sulfate_preds$sulfate <- NA
R> sulfate_with_NA <- rbind(sulfate, sulfate_preds)


We can then fit a spatial linear model by running


R> sulfmod_with_NA <- splm(
+   sulfate ^~^ 1,
+   sulfate_with_NA,
+   spcov_type = “spherical”
+ )


The missing values are ignored for model-fitting but stored in sulfmod_with_NA as newdata:


R> sulfmod_with_NA$newdata

Simple feature collection with 100 features and 1 field
Geometry type: POINT
Dimension:     XY
Bounding box:  xmin: −2283774 ymin: 582930.5 xmax: 1985906 ymax: 3037173
Projected CRS: NAD83 / Conus Albers
First 10 features:
    sulfate                  geometry
198      NA  POINT (−1771413 1752976)
199      NA   POINT (1018112 1867127)
200      NA POINT (−291256.8 1553212)
201      NA   POINT (1274293 1267835)
202      NA POINT (−547437.6 1638825)
203      NA   POINT (1445080 1981278)
204      NA  POINT (−1629090 3037173)
205      NA   POINT (1302757 1039534)
206      NA  POINT (−1429838 2523494)
207      NA   POINT (1131970 1096609)


We can then predict the missing values by running


R> predict(sulfmod_with_NA)


The call to predict() finds in sulfmod_with_NA the newdata object and is equivalent to


R> predict(sulfmod_with_NA, newdata = sulfmod_with_NA$newdata)


We can also use augment() to make the predictions for the data set with missing values by running


R> augment(sulfmod_with_NA, newdata = sulfmod_with_NA$newdata)

Simple feature collection with 100 features and 2 fields
Geometry type: POINT
Dimension:     XY
Bounding box:  xmin: −2283774 ymin: 582930.5 xmax: 1985906 ymax: 3037173
Projected CRS: NAD83 / Conus Albers
# A tibble: 100 x 3
   sulfate .fitted            geometry
 *   <dbl>   <dbl>         <POINT [m]>
 1      NA    1.40  (−1771413 1752976)
 2      NA   24.5    (1018112 1867127)
 3      NA    8.99 (−291256.8 1553212)
 4      NA   16.4    (1274293 1267835)
 5      NA    4.91 (−547437.6 1638825)
 6      NA   26.7    (1445080 1981278)
 7      NA    3.00  (−1629090 3037173)
 8      NA   14.3    (1302757 1039534)
 9      NA    1.49  (−1429838 2523494)
10      NA   14.4    (1131970 1096609)
# … with 90 more rows


Unlike predict(), augment() explicitly requires the newdata argument be specified in order to obtain predictions. Omitting newdata (e.g., running augment(sulfmod_with_NA)) returns model diagnostics, not predictions.

For areal data models fit with spautor(), predictions cannot be computed at locations that were not incorporated in the neighborhood structure used to fit the model. Thus, predictions are only possible for observations in data whose response values are missing (NA), as their locations are incorporated into the neighborhood structure. For example, we make predictions of log seal trends at the missing polygons from Fig 3 by running


R> predict(sealmod)


We can also use augment() to make the predictions:


R> augment(sealmod, newdata = sealmod$newdata)

Simple feature collection with 28 features and 2 fields
Geometry type: POLYGON
Dimension:     XY
Bounding box:  xmin: 913618.8 ymin: 1007542 xmax: 1115097 ymax: 1132682
Projected CRS: NAD83 / Alaska Albers
# A tibble: 28 x 3
   log_trend .fitted
 *     <dbl>   <dbl>
 1        NA −0.113 
 2        NA −0.0108
 3        NA −0.0608
 4        NA −0.0383
 5        NA −0.0730
 6        NA −0.0556
 7        NA −0.0968
 8        NA −0.0716
 9        NA −0.0776
10        NA −0.0647
# … with 18 more rows, and 1 more
#   variable: geometry <POLYGON [m]>


## Advanced features

spmodel offers several advanced features for fitting spatial linear models. We briefly discuss some of these features next using the moss data and some simulated data. Technical details for each advanced feature can be seen by running vignette(“technical”, “spmodel”).

### Fixing spatial covariance parameters

We may desire to fix specific spatial covariance parameters at a particular value. Perhaps some parameter value is known, for example. Or perhaps we want to compare nested models where a reduced model uses a fixed parameter value while the full model estimates the parameter. Fixing spatial covariance parameters while fitting a model is possible using the spcov_initial argument to splm() and spautor(). The spcov_initial argument takes an spcov_initial object (run help(“spcov_initial”, “spmodel”) for more). spcov_initial objects can also be used to specify initial values used during optimization, even if they are not assumed to be fixed. By default, spmodel uses a grid search to find suitable initial values to use during optimization.

As an example, suppose our goal is to compare a model with an exponential covariance and dependent error variance, independent error variance, and range parameter to a similar model that instead assumes the independent random error variance parameter (nugget) is zero. First, the spcov_initial object is specified for the latter model:


R> init <- spcov_initial(“exponential”, ie = 0, known = “ie”)
R> init
$initial
ie 
 0 

$is_known
  ie 
TRUE 

attr(,“class”)
[1] “exponential”


The init output shows that the ie parameter has an initial value of zero that is assumed to be known. Next the model is fit:


R> spmod_red <- splm(log_Zn ^~^ log_dist2road, moss, spcov_initial = init)


Notice that because the spcov_initial object contains information about the spatial covariance type, the spcov_type argument is not required when spcov_initial is provided. We can use glances() to glance at both models:


R> glances(spmod, spmod_red)

# A tibble: 2 x 10
  model         n     p  npar value   AIC  AICc logLik deviance
  <chr>     <int> <dbl> <int> <dbl> <dbl> <dbl>  <dbl>    <dbl>
1 spmod       365     2     3  367.  373.  373.  −184.     363 
2 spmod_red   365     2     2  378.  382.  382.  −189.     374.
# … with 1 more variable: pseudo.r.squared <dbl>


The lower AIC and AICc of the full model compared to the reduced model indicates that the independent random error variance is important to the model. A likelihood ratio test comparing the full and reduced models is also possible using anova().

Another application of fixing spatial covariance parameters involves calculating their profile likelihood confidence intervals [38]. Before calculating a profile likelihood confidence interval for **Θ**_*i*_, the *i*th element of a general parameter vector **Θ**, it is necessary to obtain $-2\ell (\hat{\Theta })$, minus twice the log-likelihood evaluated at the estimated parameter vector, $\hat{\Theta }$. Then a (1 − *α*)% profile likelihood confidence interval is the set of values for **Θ**_*i*_ such that $2\ell \left(\hat{\Theta }\right)-2\ell \left({\hat{\Theta }}_{-i}\right)\leq \chi_{1,1-\alpha }^{2}$, where $\ell ({\hat{\Theta }}_{-i})$ is the value of the log-likelihood maximized after fixing **Θ**_*i*_ and optimizing over the remaining parameters, **Θ**_−*i*_, and $\chi_{1,1-\alpha }^{2}$ is the 1 − *α* quantile of a chi-squared distribution with one degree of freedom. The result follows from inverting a likelihood ratio test comparing the full model to a reduced model that fixes the value of **Θ**_*i*_. Because computing profile likelihood confidence intervals requires refitting the model many times for different fixed values of **Θ**_*i*_, it can be computationally intensive. This approach can be generalized to yield joint profile likelihood confidence intervals cases when *i* has dimension greater than one.

### Fitting and predicting for multiple models

Fitting multiple models is possible with a single call to splm() or spautor() when spcov_type is a vector with length greater than one or spcov_initial is a list (with length greater than one) of spcov_initial objects. We fit three separate spatial linear models using the exponential spatial covariance, spherical spatial covariance, and no spatial covariance by running


R> spmods <- splm(
+   sulfate ^~^ 1,
+   sulfate,
+   spcov_type = c(“exponential”, “spherical”, “none”)
+ )


Then glances() is used to glance at each fitted model object:


R> glances(spmods)

# A tibble: 3 x 10
  model           n     p  npar value   AIC  AICc logLik deviance
  <chr>       <int> <dbl> <int> <dbl> <dbl> <dbl>  <dbl>    <dbl>
1 spherical     197     1     3 1137. 1143. 1143.  −569.     196.
2 exponential   197     1     3 1140. 1146. 1146.  −570.     196.
3 none          197     1     1 1448. 1450. 1450.  −724.     196 
# … with 1 more variable: pseudo.r.squared <dbl>


And predict() is used to predict newdata separately fo each fitted model object:


R> predict(spmods, newdata = sulfate_preds)


Currently, glances() and predict() are the only spmodel generic functions that operate on an object that contains multiple model fits. Generic functions that operate on individual models can still be called when the argument is an individual model object. For example, we can compute the AIC of the model fit using the exponential covariance function by running


R> AIC(spmods$exponential)

[1] 1145.824


### Random effects

Non-spatial random effects incorporate additional sources of variability into model fitting. They are accommodated in spmodel using similar syntax as for random effects in the nlme [32] and lme4 [39] R packages. Random effects are specified via a formula passed to the random argument. Next, we show two examples that incorporate random effects into the spatial linear model using the moss data.

The first example explores random intercepts for the sample variable. The sample variable indexes each unique location, which can have replicate observations due to field duplicates (field_dup) and lab replicates (lab_rep). There are 365 observations in moss at 318 unique locations, which means that 47 observations in moss are either field duplicates or lab replicates. It is likely that the repeated observations at a location are correlated with one another. We can incorporate this repeated-observation correlation by creating a random intercept for each level of sample. We model the random intercepts for sample by running


R> rand1 <- splm(
+   log_Zn ^~^ log_dist2road,
+   moss,
+   spcov_type = “exponential”,
+   random = ^~^ sample
+ )


Note that ^~^ sample is shorthand for ^~^ (1 | sample), which is more explicit notation that indicates random intercepts for each level of sample.

The second example adds a random intercept for year, which creates extra correlation for observations within a year. It also adds a random slope for log_dist2road within year, which lets the effect of log_dist2road vary between years. We fit this model by running


R> rand2 <- splm(
+   log_Zn ^~^ log_dist2road,
+   moss,
+   spcov_type = “exponential”,
+   random = ^~^ sample + (log_dist2road ^|^ year)
+ )


Note that ^~^ sample + (log_dist2road | year) is shorthand for ^~^ (1 | sample) + (log_dist2road | year). If only random slopes within a year are desired (and no random intercepts), a - 1 is given to the relevant portion of the formula: (log_dist2road—1 | year). When there is more than one term in random, each term must be surrounded by parentheses (recall that the random intercept shorthand automatically includes relevant parentheses).

We can compare the AIC of all three models by running


R> AIC(spmod, rand1, rand2)

      df      AIC
spmod  3 373.2089
rand1  4 343.1021
rand2  6 201.8731


The rand2 model has the lowest AIC.

It is possible to fix random effect variances using the randcov_initial argument, and randcov_initial can also be used to set initial values for optimization.

### Partition factors

A partition factor is a variable that allows observations to be uncorrelated when they are from different levels of the partition factor. Partition factors are specified in spmodel by providing a formula with a single variable to the partition_factor argument. Suppose that for the moss data, we would like observations in different years (year) to be uncorrelated. We fit a model that treats year as a partition factor by running


R> part <- splm(
+   log_Zn ^~^ log_dist2road,
+   moss,
+   spcov_type = “exponential”,
+   partition_factor = ^~^ year
+ )


### Anisotropy

**Fig 5:**
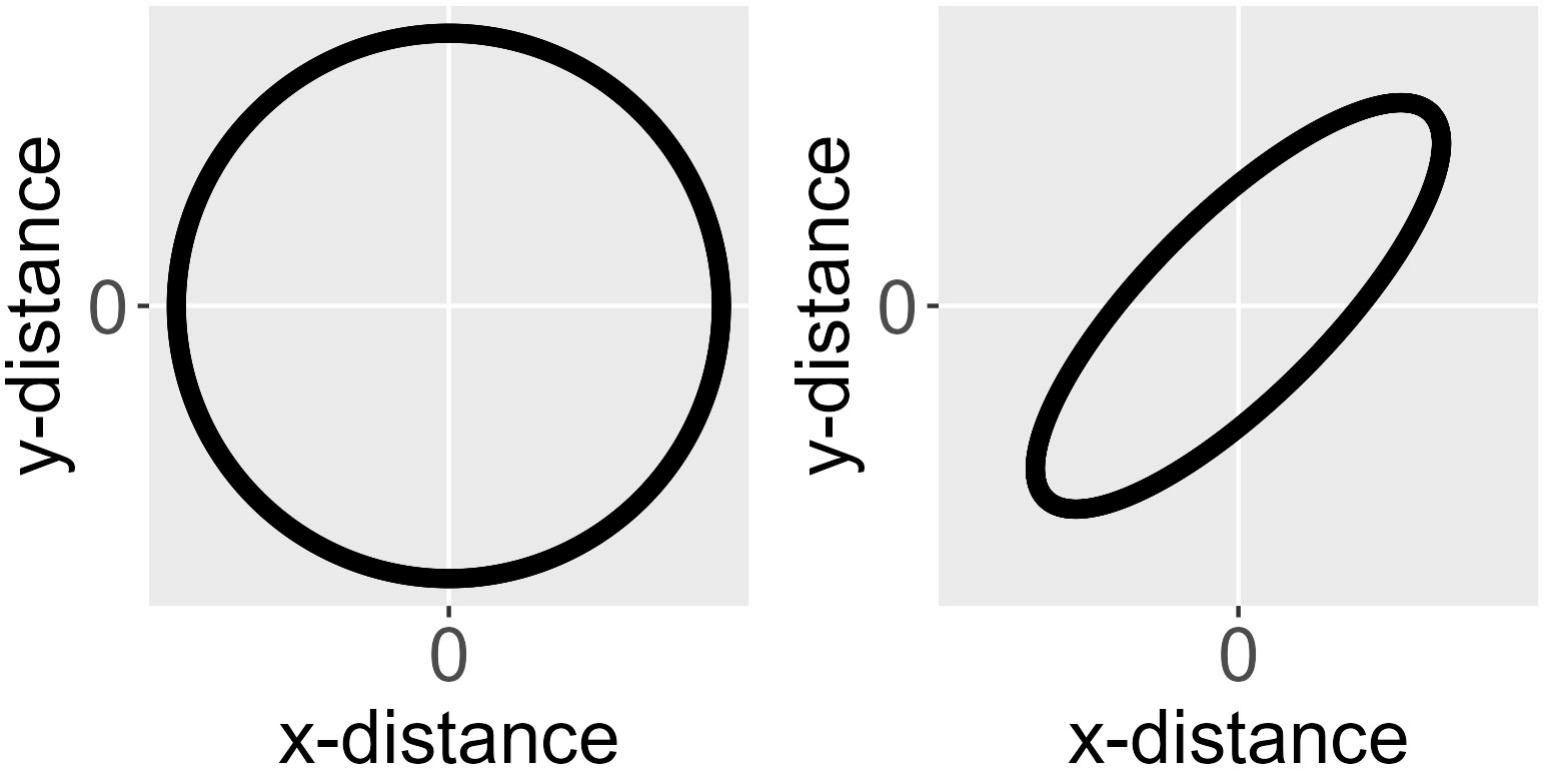
Ellipses for an isotropic and anisotropic covariance function centered at the origin. In A (left), the isotropic covariance function is visualized. In B (right), the anisotropic covariance function is visualized. The black outline of each ellipse is a level curve of equal correlation.

An isotroptic spatial covariance function (for point-referenced data) behaves similarly in all directions (i.e., is independent of direction) as a function of distance. An anisotropic covariance function does not behave similarly in all directions as a function of distance. Consider the spatial covariance imposed by an eastward-moving wind pattern. A one-unit distance in the x-direction likely means something different than a one-unit distance in the y-direction. Fig 5 shows ellipses for an isotropic and anisotropic covariance function centered at the origin (a distance of zero). The black outline of each ellipse is a level curve of equal correlation. The left ellipse (a circle) represents an isotropic covariance function. The distance at which the correlation between two observations lays on the level curve is the same in all directions. The right ellipse represents an anisotropic covariance function. The distance at which the correlation between two observations lays on the level curve is different in different directions.

Accounting for anisotropy involves a rotation and scaling of the x-coordinates and y-coordinates such that the spatial covariance function that uses these transformed distances is isotropic. We use the anisotropy argument to splm() to fit a model with anisotropy by running


R> spmod_anis <- splm(
+   log_Zn ^~^ log_dist2road,
+   moss,
+   spcov_type = “exponential”,
+   anisotropy = TRUE
+ )
R> summary(spmod_anis)

Call:
splm(formula = log_Zn ^~^ log_dist2road, data = moss, spcov_type = “exponential”, 
    anisotropy = TRUE)

Residuals:
    Min      1Q  Median      3Q     Max 
−2.5279 −1.2239 −0.7202 −0.1921  1.1659 

Coefficients (fixed):
              Estimate Std. Error z value Pr(>|z|)    
(Intercept)    9.54798    0.22291   42.83   <2e-16 ***
log_dist2road −0.54601    0.01855  −29.44   <2e-16 ***
---
Signif. codes:  0 ‘***’ 0.001 ‘**’ 0.01 ‘*’ 0.05 ‘.’ 0.1 ‘ ’ 1

Pseudo R-squared: 0.7048

Coefficients (exponential spatial covariance):
       de        ie     range    rotate     scale 
3.561e-01 6.812e-02 8.732e+03 2.435e+00 4.753e-01 
attr(,“class”)
[1] “exponential”


The rotate parameter is between zero and *π* radians and represents the angle of a clockwise rotation of the ellipse such that the major axis of the ellipse is the new x-axis and the minor axis of the ellipse is the new y-axis. The scale parameter is between zero and one and represents the ratio of the distance between the origin and the edge of the ellipse along the minor axis to the distance between the origin and the edge of the ellipse along the major axis. The transformation that turns an anisotropic ellipse into an isotropic one (i.e., a circle) requires rotating the coordinates clockwise by rotate and then scaling them the reciprocal of scale. The transformed coordinates are then used instead of the original coordinates to compute distances and spatial covariances.

Note that specifying an initial value for rotate that is different from zero, specifying an initial value for scale that is different from one, or assuming either rotate or scale are unknown in spcov_initial will cause splm() to fit a model with anisotropy (and will override anisotropy = FALSE). Estimating anisotropy parameters is only possible for maximum likelihood and restricted maximum likelihood estimation, but fixed anisotropy parameters can be accommodated for semivariogram weighted least squares or semivariogram composite likelihood estimation. Also note that anisotropy is not relevant for areal data because the spatial covariance function depends on a neighborhood structure instead of distances between locations.

### Simulating spatial data

The sprnorm() function is used to simulate normal (Gaussian) spatial data. To use sprnorm(), the spcov_params() function is used to create an spcov_params object. The spcov_params() function requires the spatial covariance type and parameter values. We create an spcov_params object by running


R> sim_params <- spcov_params(“exponential”, de = 5, ie = 1, range = 0.5)


We set a reproducible seed and then simulate data at 3000 random locations in the unit square using the spatial covariance parameters in sim_params by running


R> set.seed(0)
R> n <- 3000
R> x <- runif(n)
R> y <- runif(n)
R> coords <- tibble::tibble(x, y)
R> resp <- sprnorm(
+   sim_params,
+   data = coords,
+   xcoord = x,
+   ycoord = y
+ )
R> sim_data <- tibble∷tibble(coords, resp)


**Fig 6:**
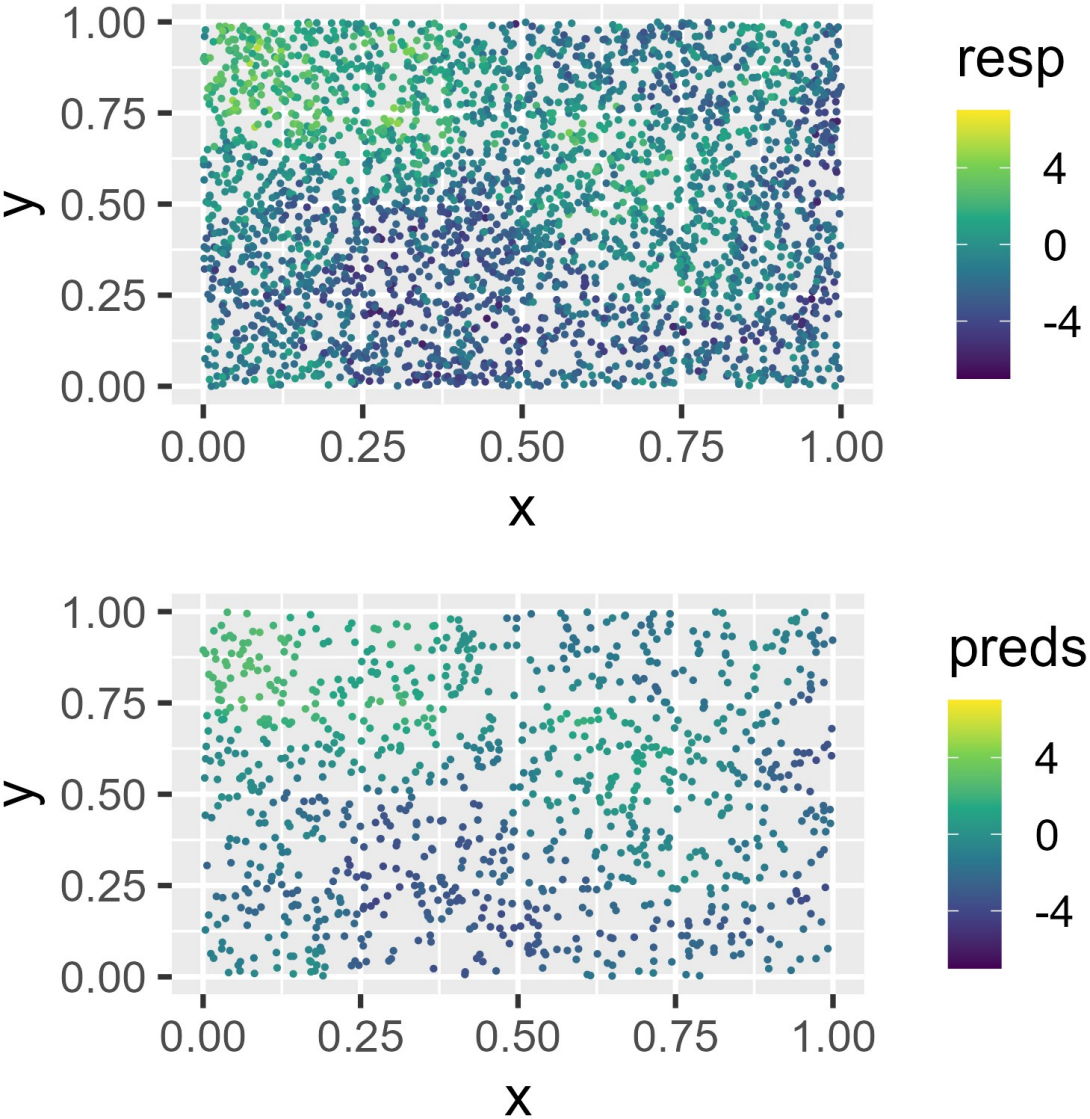
Observed data and big data predictions at unobserved locations. In A (top), spatial data are simulated in the unit square. A spatial linear model is fit using the default big data approximation for model-fitting. In B (bottom), predictions are made using the fitted model and the default big data approximation for prediction.

We can visualize the simulated data (Fig 6A) by running


R> ggplot(sim_data, aes(x = x, y = y, color = resp)) +
+   geom_point(size = 1.5) +
+   scale_color_viridis_c(limits = c(-7, 7)) + 
+   theme_gray(base_size = 18)


There is noticeable spatial patterning in the response variable (resp). The default mean in sprnorm() is zero for all observations, though a mean vector can be provided using the mean argument. The default number of samples generated in sprnorm() is one, though this can be changed using the samples argument. Because sim_data is a tibble (data.frame) and not an sf object, the columns in sim_data representing the x-coordinates and y-coordinates must be provided to sprnorm().

Note that the output from coef(object, type = “spcov”) is a spcov_params object. This is useful we want to simulate data given the estimated spatial covariance parameters from a fitted model. Random effects are incorporated into simulation via the randcov_params argument.

### Big data

The computational cost associated with model fitting is exponential in the sample size for all estimation methods. For maximum likelihood and restricted maximum likelihood, the computational cost of estimating ***θ*** is cubic. For semivariogram weighted least squares and semivariogram composite likelihood, the computational cost of estimating ***θ*** is quadratic. The computational cost associated with estimating ***β*** and prediction is cubic in the model-fitting sample size, regardless of estimation method. Typically, samples sizes approaching 10,000 make the computational cost of model fitting and prediction infeasible, which necessitates the use of big data methods. spmodel offers big data methods for model fitting of point-referenced data via the local argument to splm(). The method is capable of quickly fitting models with hundreds of thousands to millions of observations. Because of the neighborhood structure of areal data, the big data methods used for point-referenced data do not apply to areal data. Thus, there is no big data method for areal data or local argument to spautor(), so model fitting sample sizes cannot be too large. spmodel offers big data methods for prediction of point-referenced data or areal data via the local argument to predict(), capable of quickly predicting hundreds of thousands to millions of observations rather quickly.

To show how to use spmodel for big data estimation and prediction, we use the sim_data data from the previous subsection. Because sim_data is a tibble (data.frame) and not an sf object, the columns in data representing the x-coordinates and y-coordinates must be explicitly provided to splm().

#### Model-fitting

spmodel uses a “local indexing” approximation for big data model fitting of point-referenced data. Observations are first assigned an index. Then for the purposes of model fitting, observations with different indexes are assumed uncorrelated. Assuming observations with different indexes are uncorrelated induces sparsity in the covariance matrix, which greatly reduces the computational time of operations that involve the covariance matrix.

The local argument to splm() controls the big data options. local is a list with several arguments. The arguments to the local list control the method used to assign the indexes, the number of observations with the same index, the number of unique indexes, adjustments to the covariance matrix of $\hat{\beta }$, whether or not to use parallel processing, and if parallel processing is used, the number of cores.

Big data are most simply accommodated by setting local to TRUE. This is shorthand for local = list(method = “random”, size = 50, var_adjust = “theoretical”, parallel = FALSE), which randomly assigns observations to index groups, ensures each index group has approximately 50 observations, uses the theoretically-correct covariance adjustment, and does not use parallel processing.


R> local1 <- splm(
+   resp ^~^ 1,
+   sim_data,
+   spcov_type = “exponential”,
+   xcoord = x,
+   ycoord = y,
+   local = TRUE
+ )
R> summary(local1)

Call:
splm(formula = resp ^~^ 1, data = sim_data, spcov_type = “exponential”, 
    xcoord = x, ycoord = y, local = TRUE)

Residuals:
    Min      1Q  Median      3Q     Max 
−5.0356 −1.3514 −0.1468  1.2842  6.5381 

Coefficients (fixed):
            Estimate Std. Error z value Pr(>|z|)
(Intercept)   −1.021      0.699   −1.46    0.144

Coefficients (exponential spatial covariance):
    de     ie  range 
2.8724 0.9735 0.2644 


Instead of using local = TRUE, we can explicitly set local. For example, we can fit a model using k-means clustering [40] on the x-coordinates and y-coordinates to create 60 groups (clusters), use the pooled variance adjustment, and use parallel processing with two cores by running


R> local2_list <- list(
+   method = “kmeans”,
+   groups = 60,
+   var_adjust = “pooled”,
+   parallel = TRUE,
+   ncores = 2
+ )
R> local2 <- splm(
+   resp ^~^ 1,
+   sim_data,
+   spcov_type = “exponential”,
+   xcoord = x,
+   ycoord = y,
+   local = local2_list
+ )
R> summary(local2)

Call:
splm(formula = resp ^~^ 1, data = sim_data, spcov_type = “exponential”, 
    xcoord = x, ycoord = y, local = local2_list)

Residuals:
     Min       1Q   Median       3Q      Max 
−4.98801 −1.30386 −0.09927  1.33176  6.58567 

Coefficients (fixed):
            Estimate Std. Error z value Pr(>|z|)    
(Intercept)  −1.0683     0.1759  −6.073 1.25e-09 ***
---
Signif. codes:  0 ‘***’ 0.001 ‘**’ 0.01 ‘*’ 0.05 ‘.’ 0.1 ‘ ’ 1

Coefficients (exponential spatial covariance):
    de     ie  range 
2.5434 0.9907 0.2312 


Likelihood-based statistics like AIC(), AICc(), logLik(), and deviance() should not be used to compare a model fit with a big data approximation to a model fit without a big data approximation, as the two approaches maximize different likelihoods.

#### Prediction

For point-referenced data, spmodel uses a “local neighborhood” approximation for big data prediction. Each prediction is computed using a subset of the observed data instead of all of the observed data. Before further discussing big data prediction, we simulate 1000 locations in the unit square requiring prediction:


R> n_pred <- 1000
R> x <- runif(n_pred)
R> y <- runif(n_pred)
R> sim_preds <- tibble::tibble(x = x, y = y)


The local argument to predict() controls the big data options. local is a list with several arguments. The arguments to the local list control the method used to subset the observed data, the number of observations in each subset, whether or not to use parallel processing, and if parallel processing is used, the number of cores.

The simplest way to accommodate big data prediction is to set local to TRUE. This is shorthand for local = list(method = “covariance”, size = 50, parallel = FALSE), which implies that for each location requiring prediction, only the 50 observations in the data most correlated with it are used in the computation, and parallel processing is not used. Using the local1 fitted model, we store these predictions as a variable called preds in the sim_preds data by running


R> sim_preds$preds <- predict(local1, newdata = sim_preds, local = TRUE)


The predictions are visualized (Fig 6B) by running


R> ggplot(sim_preds, aes(x = x, y = y, color = preds)) +
+   geom_point(size = 1.5) +
+   scale_color_viridis_c(limits = c(-7, 7)) + 
+   theme_gray(base_size = 18)


They display a similar pattern as the observed data.

Instead of using local = TRUE, we can explicitly set local:


R> pred_list <- list(
+   method = “distance”,
+   size = 30,
+   parallel = TRUE,
+   ncores = 2
+ )
R> predict(local1, newdata = sim_preds, local = pred_list)


This code implies that uniquely for each location requiring prediction, only the 30 observations in the data closest to it (in terms of Euclidean distance) are used in the computation and parallel processing is used with two cores.

For areal data, no local neighborhood approximation exists because of the data’s underlying neighborhood structure. Thus, all of the data must be used to compute predictions and by consequence, method and size are not components of the local list. The only components of the local list for areal data are parallel and ncores.

## Discussion

spmodel is a novel, relevant contribution used to fit, summarize, and predict for a variety of spatial statistical models. Spatial linear models for point-referenced data (i.e., geostatistical models) are fit using the splm() function. Spatial linear models for areal data (i.e., autoregressive models) are fit using the spautor() function. Both functions use a common framework and syntax structure. Several model-fit statistics and diagnostics are available. The broom functions tidy() and glance() are used to tidy and glance at a fitted model. The broom function augment() is used to augment data with model diagnostics and augment newdata with predictions. Several advanced features are available to accommodate fixed covariance parameter values, random effects, partition factors, anisotropy, simulating data, and big data approximations for model fitting and prediction.

We appreciate feedback from users regarding spmodel, and we have several plans to add new features to spmodel in the future. To learn more about spmodel or provide feedback, please visit our website at https://usepa.github.io/spmodel/.
