# Supplementary material for: spmodel: Spatial statistical modeling and prediction in R
Source: PLoS One. 2023 Mar 9;18(3):e0282524. doi: 10.1371/journal.pone.0282524 (PMC9997982; doi:10.1371/journal.pone.0282524)
Supplement: Attachment — Submitted filename: review_response.pdf [file pone.0282524.s001.pdf]

Review responses for "spmodel: spatial statistical modeling and prediction in **R**"

Michael Dumelle [1] [*], Matt Higham [2] , Jay M. Ver Hoef [3]

1 United States Environmental Protection Agency, Corvallis, Oregon, United States of America
2 St. Lawrence University Department of Math, Computer Science, and Statistics, Canton, New York, United States of America
3 National Oceanic and Atmospheric Administration Alaska Fisheries Science Center, Marine Mammal Laboratory, Seattle, Washington, United States of America

* Corresponding author: Dumelle.Michael@epa.gov

Preface

We would like to sincerely thank the academic editor, A. K. M. Anisur Rahman, Ph.D., and the two anonymous reviewers for their hard work and time spent providing us with this thoughtful, valuable feedback that greatly improved the manuscript. We have addressed each comment below, starting with reviewer 1 and then for reviewer 2. Original reviewer comments are indicated by a dot and responses are indicated by a dash. This structure can be seen below

- Reviewer comment
    - Response to comment

We have addressed all reviewer comments in this document and look forward to resubmitting the manuscript. If there is any dissatisfaction regarding the way we addressed specific comments, please let us know and we will revise appropriately.

Reviewer 1

- Data are always plural. Change "data is" to "data are" throughout, with the exception of the object named "data".
    - We have incorporated this change throughout.
- Introduction: Suggest mentioning INLA in review of R packages used for modelling point-referenced and areal data. I believe INLA and rstan can be used to model both, but in both cases the unique object structure and syntax leads to a fairly significant learning curve for new users.
    - We have added citations for `R-INLA` [1], `rstan` [2], `brms` [3],
- Lines 238-239: I suggest reminding the reader that both models are fit using the default estimation method, REML, and so AIC and AICc are both valid for model comparison. Alternatively, you could add the estmethod = "reml" to the function call to make it more obvious.

  - We have added the following sentence at the end of the referenced paragraph: “Recall that these AIC and AICc comparisons are valid because both models are fit using restricted maximum likelihood (the default).”

- Line 271: This sentence about leverage feels incomplete compared to the explanation given to other functions and outputs. I suggest adding something to the effect of “large leverage values indicate that an observation may be an outlier and warrant further investigation”, without going into too much detail. Later I see that leverage is mentioned again in lines 302-303 and Cooks D vs leverage is plotted. Another alternative is to add a sentence here telling readers how to interpret the plot.

  - We have adjusted the relevant sentence so it now reads: “Larger hat values indicate more leverage, and observations with large hat values may be unusual and warrant further investigation.” A similar change was made for an analogous sentence about Cook’s distance.

- Lines 274-276: I suggest including an example call to help here, which users will need to do if they’d like to change the type in the fitted function. I had trouble figuring out that the function was named fitted.spmod. I may have missed it in the help file and manuscript, but I don’t see where it says the output of splm is an object of class “spmod” – that would have helped here.

  - We agree that there are several situations where this type of information would be helpful, so we added the following few sentences to the introduction: “`spmodel` contains various methods for generic functions defined outside of `spmodel`. To find relevant documentation for these methods, run `help("generic.spmodel", "spmodel")` (e.g., `help("fitted.spmodel", "spmodel")`, `help("summary.spmodel", "spmodel")`, `help("plot.spmodel", "spmodel")`, `help("predict.spmodel", "spmodel")`, `help("tidy.spmodel", "spmodel")`, etc.) . We provide more details and examples regarding these methods and generics throughout this vignette. For a full list of `spmodel` functions available, see `spmodel`’s documentation manual.” Note that it is a new convention in `spmodel` v0.3.0 to use the `spmodel` suffix for documentation. If this resubmission is accepted, we will submit v0.3.0 to CRAN immediately so that the CRAN version aligns with this manuscript.

- Line 296, 317, 388, 399, 558, 647, 697: Unindent so that it is part of the previous paragraph.

  - We have incorporated this change throughout (and in several other places we noticed unindentation was needed).

- Lines 412-443. – output from spautor: It would be helpful to add a sentence or three about the output. For example, where is the autocorrelation parameter estimate? I noticed that the pseudo.r.squared is 0. Am I correct in my interpretation that this model has a really poor model fit? Or does it return 0 because there are no covariates in the model?

  - We have added a `summary()` call to show the autocorrelation parameter estimate. We have added the following sentence at the end of the paragraph to make the pseudo R-squared point more clear: “Note that the pseudo R-squared is zero because there are no explanatory variables in the model (i.e., it is an intercept-only model).”

- Lines 471-478: And the same factor levels if variables are factors?
  - The levels of the variable in `newdata` must be a subset of the levels in `data`. We have added the following two sentences to elucidate: "Additionally, if an explanatory variable is categorical or a factor, the values of this variable in `newdata` must also be values in `data` (e.g., if a categorical variable with values `"A"`, and `"B"` was used to fit the model, the corresponding variable in `newdata` cannot have a value `"C"`)."
- Predict function: It looks like the results are returned in different formats (vector, list), depending on the se.fit and interval arguments. It would be worthwhile to mention this here so that people know to refer to the help file, which explains this clearly. I also suggest including the example with augment (lines 495-500) in the help file, as well as the manuscript. It's really helpful.
  - We have added the following sentence referring readers to the help file: "The `predict()` output structure changes based on `interval` and `se.fit`. For more details, run `help("predict.spmodel", "spmodel")`." We also now include an example of `augment()` in the `predict()` examples section of the help file.
- Line 667: then is used twice in this sentence – awkward.
  - We have removed one usage of then. We have adjusted the relevant sentence so it now reads: "Then a $(1-\alpha)\%$ profile likelihood confidence interval is the set of values for $\mathbf{\Theta}_i$ such that $2\ell(\hat{\mathbf{\Theta}}) - 2\ell(\hat{\mathbf{\Theta}}_{-i}) \leq \chi^2_{1,1-\alpha}$, where $\ell(\hat{\mathbf{\Theta}}_{-i})$ is the value of the log-likelihood maximized after fixing $\mathbf{\Theta}_i$ and optimizing over the remaining parameters, $\mathbf{\Theta}_{-i}$, and $\chi^2_{1,1-\alpha}$ is the $1-\alpha$ quantile of a chi-squared distribution with one degree of freedom."
- Random Effects section: I really appreciate all of the examples the authors included and the additional descriptions they've provided about how to set up random effects. This is going to be very helpful for users who are either unfamiliar with R or come from a non-statistical background.
  - Thank you for the kind words!
- Line 724: Incomplete sentence.
  - We have adjusted the relevant sentence so it now reads: "The `rand2` model has the lowest AIC."
- Line 810: typo – "and" is repeated twice.
  - We have adjusted the relevant sentence so it now reads: "The `spcov_params()` function requires the spatial covariance type and parameter values."
- Lines 869-884: Are the indices mentioned in lines 869-871 used to define the groups mentioned in lines 880-883? I assume that the local parameter list defines the groups and the indices are the labels?
  - We have adjusted the relevant sentence so it now reads: "This is shorthand for `local = list(method = "random", size = 50, var_adjust = "theoretical", parallel = FALSE)`, which randomly assigns observations to index groups, ensures each index group has approximately 50 observations, uses the theoretically-correct covariance adjustment, and does not use parallel processing."

Reviewer 2

- Redaction style: Clearly, the manuscript is written following the style of a paper submitted to "Journal of Statistical Software" or "The R Journal". I am not member of the Editorial Board of PLOS ONE, so I do not see myself qualified to judge whether the current style of the manuscript is appropriate to be published in this journal.

  - This review comment is not applicable to us, though we do note that PLOS One has published several manuscripts of this nature (i.e., manuscripts that detail an R package and interweave text and code).

- Introduction: Very few references are included in the first part of the introduction section and most of them are packages/papers written by the authors of the present manuscript. Please, include additional references to spatial random sampling and statistical analysis of spatial data (as for example, [4]).

  - We have rewritten this paragraph. We decided to omit a discussion about the analysis of design-based spatial data, as it seems to distract from the focus of the manuscript, which is describing model-based inference. Thus we have removed the relevant citations written by the manuscript authors and include a reference to a discussion about model-based vs design-based inference for spatial data. We then added multiple references, including the one suggested. The relevant paragraph now reads: "`spmodel` implements model-based inference, which relies on fitting a statistical model. Model-based inference is different than design-based inference, which relies on random sampling and estimators that incorporate the properties of the random sample [5]. [6] defines two types of spatial data that can be analyzed using model-based inference: point-referenced data and areal data (areal data are sometimes called lattice data). Spatial data are point-referenced when they are observed at point-locations indexed by x-coordinates and y-coordinates on a spatially continuous surface with an infinite number of locations. Spatial models for point-referenced data are sometimes called geostatistical models. Spatial data are areal when they are observed as part of a finite network of polygons whose connections are indexed by a neighborhood structure. For example, the polygons may represent counties in a state who are neighbors if they share at least one boundary. Spatial models for areal data are sometimes called spatial autoregressive models. For thorough overviews model-based inference in a spatial context, see [6], [4], and [7]."

- Introduction: When reviewing the existing R packages to analyze and estimate areal data, I suggest the authors to include also the "diseasemapping" [8] and "bigDM" [9] packages. Additional packages for disease mapping and areal data analysis can be found in the CRAN Task View "Analysis of Spatial Data" (https://cran.r-project.org/web/views/Spatial.html)

  - We have added the "bigDM" citation [9]. The "diseasemapping" package unfortunately has been archived recently, so we have omitted that citation.

- Perhaps the authors want to update the manuscript by including some of the new features from the current version (0.2.0) of the "spmodel" package.

  - We have added an additional subsection in the "Advanced Features" section titled "Fitting and Predicting for Multiple Models" that details a main

addition of v0.2.0 of `spmodel` – fitting and predicting for multiple models at once.

References

1. Lindgren F, Rue H. Bayesian spatial modelling with r-inla. Journal of statistical software. 2015;63: 1–25.

2. Stan Development Team. RStan: The R interface to Stan. 2023. Available: https://mc-stan.org/

3. Bürkner P-C. Brms: An r package for bayesian multilevel models using stan. Journal of statistical software. 2017;80: 1–28.

4. Banerjee S, Carlin BP, Gelfand AE. Hierarchical modeling and analysis for spatial data. Chapman; Hall/CRC; 2003.

5. Brus D, De Gruijter J. Random sampling or geostatistical modelling? Choosing between design-based and model-based sampling strategies for soil (with discussion). Geoderma. 1997;80: 1–44.

6. Cressie N. Statistics for spatial data. John Wiley & Sons; 1993.

7. Schabenberger O, Gotway CA. Statistical methods for spatial data analysis: Texts in statistical science. Chapman; Hall/CRC; 2017.

8. Brown PE. Model-based geostatistics the easy way. Journal of Statistical Software. 2015;63: 1–24.

9. Adin A, Orozco-Acosta E, Vicente G, Ugarte MD. BigDM: Scalable bayesian disease mapping models for high-dimensional data. 2022. Available: https://github.com/spatialstatisticsupna/bigDM
