# Supplementary material for: spmodel: Spatial statistical modeling and prediction in R
Source: PLoS One. 2023 Mar 9;18(3):e0282524. doi: 10.1371/journal.pone.0282524 (PMC9997982; doi:10.1371/journal.pone.0282524)
Supplement: Attachment — Submitted filename: review_response.pdf [file pone.0282524.s002.pdf]

Review responses (second round) for “spmodel: spatial statistical modeling and prediction in **R**”

Michael Dumelle [1] [*], Matt Higham [2] , Jay M. Ver Hoef [3]

1 United States Environmental Protection Agency, Corvallis, Oregon, United States of America
2 St. Lawrence University Department of Math, Computer Science, and Statistics, Canton, New York, United States of America
3 National Oceanic and Atmospheric Administration Alaska Fisheries Science Center, Marine Mammal Laboratory, Seattle, Washington, United States of America

* Corresponding author: Dumelle.Michael@epa.gov

Preface

We would like to sincerely thank the academic editor, A. K. M. Anisur Rahman, Ph.D., and the two anonymous reviewers for their hard work and time spent providing us with this thoughtful, valuable feedback that greatly improved the manuscript. We have addressed each comment below, starting with reviewer 1 and then for reviewer 2. Original reviewer comments are indicated by a dot and responses are indicated by a dash. This structure can be seen below

- Reviewer comment
    - Response to comment

We have addressed all reviewer comments in this document and look forward to resubmitting the manuscript. If there is any dissatisfaction regarding the way we addressed specific comments, please let us know and we will revise appropriately.

Reviewer 1

Reviewer 1 did not suggest any changes, remarking that “all comments [from the previous review] have been addressed”.

Reviewer 2

- Page 2, first paragraph: Please, correct the name of the R-INLA package (instead of R-inla) and use the same letter font for the bigDM package.
    - We have incorporated these changes.
- References section: Check the capital letters in the titles/journal names of some references such as [11], [16], or [40].
    - We have updated our reference section to ensure proper capitalization in titles/journal names of references.
